# The case for not masking away repetitive DNA

**DOI:** 10.1186/s13100-018-0120-9

**Published:** 2018-05-01

**Authors:** R. Keith Slotkin

**Affiliations:** Department of Molecular Genetics and Center for Applied Plant Sciences, The Ohio State University, 500 Aronoff Laboratory, 318 West 12th Ave, Columbus, Ohio 43210 USA

**Keywords:** Repeat mask, Filter, Transposable element, Repetitive DNA, Transposon, Genomics

## Abstract

In the course of analyzing whole-genome data, it is common practice to mask or filter out repetitive regions of a genome, such as transposable elements and endogenous retroviruses, in order to focus only on genes and thus simplify the results. This *Commentary* is a plea from one member of the *Mobile DNA* community to all gene-centric researchers: *please do not ignore the repetitive fraction of the genome*. Please stop narrowing your findings by only analyzing a minority of the genome, and instead broaden your analyses to include the rich biology of repetitive and mobile DNA. In this article, I present four arguments supporting a case for retaining repetitive DNA in your genome-wide analysis.

## Main text

From the time of their discovery by McClintock, transposable elements (TEs) have had a history of being ignored [1]. Termed “*controlling elements*” by McClintock for their roles in developmental regulation, TEs were first disregarded, then relegated as an oddity, and are now largely overlooked by most researchers. Today, most biologists believe mobile DNA represents filler (or even worse: “junk”) between exons or genes. In single-locus experiments, this bias is propagated by alignment tools such as BLAST that mask low complexity and repetitive regions as a default option. In genome-wide experiments, even though data from all regions should be captured, this blindness to repetitive DNA is exacerbated due to the use of standard bioinformatic pipelines that restrict the analysis to genes. “Whole-genome” datasets are routinely restricted to a concentrated analysis of 22-51% of the human genome corresponding to the non-repetitive portion defined by *RepeatMasker* [2]. For example, in RNA-seq experiments researchers commonly only consider reads aligned to defined genic exons. This leaves 78-49% of the genome unanalyzed in favor of a simplified evaluation of gene expression.

This *Commentary* is not written for the existing readership of *Mobile DNA*, who already appreciate the many roles of TEs and endogenous retroviruses (ERVs). In fact, many of us in the *Mobile DNA* field have enriched our careers by making discoveries from reanalyzing publicly available data in which the original authors did not pay attention to the repetitive fraction of their data. For example, one of the most important discoveries from my own career came from reanalyzing available microarray data for TE expression [3]. Instead, it is my hope that this article is found by researchers when they perform web searches on how to filter out repetitive DNA. This *Commentary* is written by one *Mobile DNA* researcher on behalf of the field as an appeal to the general scientific community: By focusing on only a fraction of the genome, only a fraction of discoveries can be made. Below are four distinct arguments to convince the reader to retain the repetitive portion of the genome in their experiments to perform a true “genome-wide” analysis.

## Repetitive DNA critically influences gene and genome regulation

The influence of TEs and ERVs on gene expression is no longer just an oddity of a few examples of meta-stable phenotypes or epialleles. Mobile DNA is now understood to have *bona fide* genome-wide gene regulatory abilities through a number of different mechanisms, including *cis* effects on neighboring genes, and *trans*-effects at a distance. Repetitive and mobile DNA provide a rich source of gene regulation, evolutionary flexibility and epigenetic catalysts. Although too many examples exist to fit into this short *Commentary* article, I point the reader to thorough review articles on this topic (reviewed in [4, 5]). The dynamic mobile nature of TEs and ERVs, in addition to their interaction with a number of host proteins, provide the cell with a DNA-protein interaction module that if transposed near a gene, may negatively or positively affect the regulation of that gene. If positive and selected for, this module may be evolutionarily coopted as a new enhancer element. TEs and ERVs are now known to regulate single genes as well as entire gene regulatory networks [6, 7]. Therefore, if a researcher were performing a ChIP-seq experiment to identify where a transcription factor binds (for example), or a chromosome confirmation capture (4C/HiC) experiment to identify enhancer-promoter interactions, they would potentially miss important enhancers if repetitive DNA was filtered from their analysis.

In addition to their function in gene regulation, TEs and ERVs can act as developmental and tissue stage-specific markers (such as in early mammalian development [8, 9]), as sources of long non-coding RNAs [10] and as sensors of cellular stress [11]. TE-derived proteins are also involved in centromere function [12, 13], and TEs can substitute as telomeres in the absence of telomerase [14]. Importantly, TEs and ERVs are both associated with (and could be the primary cause of) several diseases [15–17]. Therefore, the message is clear: Repetitive DNA such as TEs and ERVs should not be excluded from genome-wide analyses because they play key roles in gene regulation and evolution.

## Technology has advanced to better assay the repetitive portion of the genome

Because of their limited sequenced read length, deep sequencing technologies such as Illumina SBS have fundamentally superior performance for single-copy genic regions compared to repetitive DNA. This problem is most severe for small RNA biology or when only a sequence tag can be obtained, as a large fraction of the reads cannot be mapped to a unique position in the genome because only 15-30 nucleotides of sequence information is available. However, even with limited sequence length, approaches have been developed to successfully assay the repetitive fraction of a genome with short reads (for example, [18]).

Many researchers simply and completely filter out repetitive DNA from their analysis, even though read mapping technologies have evolved or have been specifically designed to efficiently assay repetitive DNA from whole genome data. These tools include MELT, RetroSeq, EpiTEome and McClintock for TE insertion site identification [19–22], T-lex to identify presence/absence of TE copies [23], Clari-TE to resolve nested TE structure [24], TEtoolkit/TEtranscripts for TE enrichment analysis from experiments such as RNA-seq and ChIP-seq [25, 26], and many others. Which tool to use is specific to the desired type of analysis and biological question. Nevertheless, several overarching guidelines should be considered when mapping sequenced reads to repetitive DNA. First, the location in the genome where the read matches *best* should be the only one reported; however multiple *best*-matching regions may exist (i.e. multi-mapping reads). A conservative approach is to report only the reads that perfectly and uniquely match once in the genome (for example, my lab used this method for MethylC-seq of TEs in [27]). This approach should be used if the researcher wants to be absolutely sure that a particular locus is generating the reads. The problem with this approach is that it disregards a substantially large proportion of reads, especially in highly repetitive (TE-rich) genomes. Rather than having to re-map reads using different approaches based on the number of mismatches (mapping only perfect matches, then 1-mismatch, then 2, …), one can perform an intensive mapping of all possibilities at once, and use downstream tools like *NGSUtils* [28] to parse their desired level of matching preciseness from the output file. A second approach is to randomly divide the multi-mapping reads to their *best* positions in the genome (for example, my lab used this method mapping small RNA reads in [29]), although this will dilute the analysis of individual repetitive elements across their entire repeat families. This approach should be used in TE-rich genomes, and when analysis of TE families is performed but resolution down to individual elements is not necessary. A third approach is to hierarchically distribute multi-mapping reads guided by the evidence of where the uniquely mapping reads cluster [30] (for example, this method was used when mapping small RNA reads in [31]). The idea behind this approach is that we already know that some individual TE elements generate reads (by using the uniquely mapping reads), so it is most likely that these same TEs are also the producers of the multi-mapping reads. This approach should be used when TE regulation requires drilling down beyond the repeat family level to individual elements. The drawback to this approach is it has the potential to falsely assign one or more reads to a locus that did not produce them. Lastly an approach that has been utilized for years within the *Mobile DNA* community, reads can be mapped to single elements or databases of ‘consensus’ sequences that are created from alignments of many individual repetitive genomic sequences [32] (for example, my lab used this method in Figure 1-2 of [33]). This approach is useful when a family-level (rather than an individual element) view is required in high detail. All of the above mentioned approaches (with the strong exception of not interrogating the repetitive fraction of the genome at all) are acceptable to the *Mobile DNA* community, given that the researcher is transparent about which approach was used.

In contrast to software advances, the key enabling technology that has enhanced the read mappability (and therefore coverage) of the repetitive fraction of the genome is improved read length. On the widely-used Illumina SBS platform, the standard read length has transitioned from 50 to 150 nucleotides, and when coupled with a paired-end sequencing and mapping approach, coverage of the non-genic portion of the genome has greatly improved. The significantly longer reads of PacBio and Nanopore sequencing approaches altogether remove the mapping ambiguity that has previously limited the genome-wide analysis of repetitive DNA. For many experiments, extending the length of the sequence read is already worth the investment to enable unique alignment to the genome, and the future will see these technologies used routinely. Long read lengths also enable improved split-read mapping approaches to identify non-reference genome positions of mobile TEs [21, 34]. Therefore, the read length and mapping approaches described here now exist to at least partially, if not all together, cover the repetitive fraction of the genome, and thus interrogating repetitive DNA should be a necessary component of any “genome-wide” analysis.

## Many technologies have originated from mobile DNA or defenses against them

Basic research performed on mobile DNA aims to identify how elements transpose, the mechanisms responsible for repressing activity, and the short-term and evolutionary consequences of transposition/insertion. Medical applied research also aims to identify how TEs and ERVs contribute to cancer, neurological disease and other disorders. In addition to these areas of focus, the investigation of mobile DNA has significantly contributed to the production of new research tools. Mobile DNA biology has enabled improved mutagenesis and gene tagging, transgenesis, and analysis of gene function by gene trapping, just to name a few [35]. In vitro TE systems have been leveraged to generate protocols to assay chromatin accessibility (ATAC-seq) [36] as well as efficient deep sequencing library production (tagmentation) [37]. Even the key enabling technology of this decade, CRISPR-Cas genome editing, is now understood to be based upon mobile DNA [38]. Therefore, paying attention to the analysis of repetitive DNA, even on the basic research level, may pay dividends towards the development of a key technological breakthrough that can propel multiple disciplines forward.

## Maximize the data output from your effort (and cost) input

It takes a substantial amount of effort to execute (and fund) research science. Once the funds are successfully obtained, and the experiments are performed, it simply does not make sense to limit the analysis to a fraction of the obtained data. Confining an analysis to only part of the genome significantly restricts the likelihood and scope of the ensuing discoveries. Two recent examples of TE/ERV analyses done well both represent cases where considering repetitive elements answered questions that would otherwise be unexplainable: 1) The structural variation map of 2504 genomes that specifically identified *Alu*, L1 and SVA mobile element insertions across human populations [39] and 2) a ChIP-seq analysis discovered that ERVs have spread interferon-inducible enhancer elements and shaped the evolution of innate immunity [7].

Many published datasets exist that can be downloaded and for the first time analyzed for TE or ERV dynamics. The availability of this only partially analyzed data affords great opportunity to my colleagues in the *Mobile DNA* field; however, the analysis of this data for repetitive DNA dynamics should have been part of the original study. We, the *Mobile DNA* Community, request that you please include repetitive DNA in your genome-wide analyses. If you do not have the tools or the interest, contact a *Mobile DNA* community member in your field to collaborate. Because, after all, if you don’t analyze the “whole genome” from your genome-wide data, we will.

## Abbreviations

ATAC-seq: Assay for transposase-accessible chromatin using sequencing
ERV: Endogenous retrovirus
HiC/4C: Chromosome confirmation capture experiments
SBS: Sequence by synthesis
TE: Transposable element
